# Misalignments in athletic ambitions and motives for participation in youth football: a cross-sectional study of players, parents, and coaches

**DOI:** 10.3389/fspor.2026.1769158

**Published:** 2026-02-26

**Authors:** Siv Skard, Malene Joensen, Hilde Gundersen, Hege Randi Eriksen

**Affiliations:** 1 Department of Strategy and Management, Norwegian School of Economics, Bergen, Norway; 2 Department of Sport, Food and Natural Sciences, Western University of Applied Sciences, Bergen, Norway

**Keywords:** ambition, football, gender, motives, parents, sport, youth

## Abstract

**Introduction:**

While the gender gap in ambitions and motives in sports has been widely studied, less attention has been paid to the potential discrepancies between the perspectives of youth athletes, their coaches, and their parents. This study examines the extent to which youth football players' ambitions and motives for participation are accurately perceived by two key socializers: coaches and parents. Furthermore, it investigates whether systematic gender differences exist in these perceptions.

**Methods:**

Utilizing a cross-sectional survey, we collected data from youth football players, age 13–16 yrs (*N* = 593, 44.8% female), their coaches (*N* = 99, 11.1% female), and their parents (*N* = 528, 59.8% female).

**Results:**

Our findings reveal significant misalignments between the views of players, coaches, and parents regarding the players' level of ambition and main motives to participate in organized football. Females' motives and ambitions are in particular underestimated by their parents and coaches. These misalignments could reinforce gendered narratives in sports, potentially leading to unequal resource allocation.

**Discussion:**

Our study highlights the need for better alignment between the perceptions of coaches and parents and the actual ambitions and motives among youth players to promote equal support and development opportunities for all.

## 1 Introduction

Prior research has investigated gender differences in youths' athletic ambitions and motivational drivers for participating in sports. This research often suggests that females exhibit lower athletic ambitions (1), are less motivated by the prospect of becoming a professional athlete (2), and are more strongly motivated by social factors (3). Moreover, female athletes tend to be more task-oriented, focusing on skill improvement and mastery, while male athletes are generally more ego-oriented, driven by performance and results (4–7). However, regardless of any actual gender differences, gendered misconceptions among key socializers (e.g., parents and coaches) may shape how youths' motives and ambitions are interpreted, potentially producing perceived gender differences that are overstated or not present in athletes' self-reports. The present study examines such misalignments by comparing athletes' self-reports with parents' and coaches' perceptions.

While acknowledging the complexity of explanatory factors contributing to gender differences in motivation and ambitions, one possible explanation is the culturally embedded narrative about gender in sport. In many sports, stereotypical masculine traits tend to be over-emphasized (8), creating a narrative about sports that marginalizes girls and women (2). Skewed media attention towards male athletes (9) contributes to this narrative and creates an environment where girls lack female athletic role models (10). According to Social Role Theory (11), the underrepresentation of female athletes in the media influences societal perceptions regarding the appropriateness of women as athletes. The gender difference in motives for participating in sport is likely to have been influenced by what society regards as proper behavior for men and women (3).

In a society where sport is stereotyped as masculine and female athletes are underrepresented in the media, it is likely that there are gendered misconceptions about young athletes' true motivations and ambitions. While several studies have focused on gender differences in motivation in sports, a critical question that remains unexplored is whether males' and females' motives for participation in youth sport are accurately perceived by their coaches and parents. Some research has explored discrepancies between coaches' priorities and athletes' motivations. For instance, Zarrett et al. (12) compared the motivations of female youth athletes with their coaches' motivations, revealing that female athletes prioritize competitiveness and skill development as key aspects of having fun with their sport, whereas coaches tend to de-emphasize competitiveness. However, no research to date has systematically compared youth athletes' self-reported motives and ambitions in sport with the perceptions held by both their coaches and parents.

This gap is important because inaccurate perceptions—whether underestimating or overestimating motives—can lead to biased support and encouragement, ultimately shaping the opportunities available to young athletes. For example, research indicates that having fun and socializing with friends are primary motivations for participation among both boys and girls (13). Yet gender biases may lead parents and coaches to overestimate girls' social motivations and underestimate their athletic ambitions. Since competitiveness is often stereotyped as a masculine trait (3), there is a risk that socializers may overestimate achievement motives and ambitions for boys, while underestimating them for girls. Conversely, if girls are presumed to be less ambitious, parents and coaches may overemphasize hedonic motives (e.g., fun and socializing) rather than fostering competitive or achievement-oriented aspirations.

Gendered narratives in sports are sustained by a complex ecosystem of various actors and significant socializers, each potentially contributing to the reinforcement of stereotypes and discriminatory practices. Eccles' expectancy-value theory (14) provides a framework for understanding the role of socializers' beliefs and behaviors in shaping motivations in achievement settings. This theory has been applied to explain how parental socialization practices influence children's motivation in sport (15). A central prediction is that children tend to adopt their parents' beliefs, meaning that their own motivations and expectations of success will resonate with “a belief system that originated with their parents” (16). Therefore, parents' gender-stereotypical beliefs about their sons' and daughters' participation in sports are likely to shape the feedback they provide, which in turn may reinforce gender stereotypes within the children's own belief systems. Parents who believe that males have superior athletic competencies compared to females will tend to provide better opportunities and encouragement for their sons than they do for their daughters. The difference in parents' socialization practices based on gender will influence the children's beliefs about their own athletic abilities (15).

Work by Eccles and colleagues (15, 17, 18) has extensively demonstrated that parents' expectations influence children's motivations and performance. In the context of sports, coaches also serve as critical socializing agents for young athletes. While Weltevreden et al. (19) found that parents have a greater influence on children's achievement goals than coaches do, coaches will arguably also play an important role in athletes' sports experiences (36), as they influence players' motivation (20, 21) and their enjoyment of the sport (22).

The purpose of this study is to identify potential misalignments between the motives and ambitions reported by youth football players and the perceptions held by their parents and coaches. We test two overarching hypotheses:

**H1:** Parents' and coaches' perceptions will differ from players' self-reported motives and ambitions.
**H2:** These misalignments will be more pronounced for female players.

This research is set in Norway, where organized youth sport is publicly funded through the national umbrella organization, the Norwegian Olympic and Paralympic Committee and Confederation of Sports, which operates under the vision “Joy of Sport for All” (23). Organized sport is part of the civic sector and relies heavily on volunteer work (24). Approximately 78% of all coaches in Norwegian sport are volunteers, many of whom are parents of children participating in sport (25). Football is the most popular organized sport for both males and females, with a female participation rate of 31% (26). Despite its evident appeal to young female athletes, football is stereotyped as a masculine sport, and Norwegian clubs invest significantly more resources in developing male talents than female talents (27). This context makes Norwegian youth football a particularly relevant case for examining these issues.

## 2 Material and methods

### 2.1 Sampling procedures and participants

The target populations for this study were Norwegian youth football players (age 13–16), their coaches, and their parents. Players of this age span corresponds to the transition into “youth football” in Norwegian organized football (28). It is an age group well suited for questionnaire-based measurement, since early adolescents can generally provide reliable self-reports using standardized response scales. This period is also relevant because it marks a developmental transition in sport participation where motivations, ambitions, and socializer influence are salient.

There are 18 regional football confederations in Norway. Since the collaborators on the research project are in the Hordaland County, we sampled from this confederation. Hordaland has the 3rd largest regional football confederation in Norway, consisting of 217 youth girls' teams and 450 youth boys' teams at the time of this research.

The sampling procedures were as follows: Hordaland Football Confederation provided a list of all clubs in the region that had youth teams for males and/or females. These clubs varied in geographical location (urban or rural areas), size (number of members), and quality club status (a designation based on various criteria set by the confederation). A representative from the confederation, blind to the research questions, was tasked with selecting 50 clubs to receive an invitation to participate in the study. The clubs were selected to reflect the distribution of quality club status (approx. 30%) and include variation in geographical location and club size. Although boys are overrepresented in the population, we aimed for an equal gender distribution to align with the gender focus in this study.

A total of 33 teams (51.5% female teams; 45.5% quality clubs) accepted the invitation and participated in the survey. We excluded 5 players who did not disclose their gender. The final sample included 593 players (44.8% female), 528 parents (59.8% female) and 99 coaches (11.1% female).

### 2.2 Procedures

Teams from the 50 invited clubs received an e-mail invitation to participate in a digital survey about youth football, targeting players, their coaches, and their parents. The Hordaland Football Confederation sent the invitation, informing that the survey was part of a research project conducted by the local university. Participation was incentivized by an invitation to a social event for players and their coaches, where food would be served. This event was funded by the project's corporate partner, Sparebanken Vest, the local savings bank, but organized by the clubs at their respective locations. To ensure high-quality data and completion rate, players and coaches of the selected teams completed the digital survey, hosted on Qualtrics (Qualtrics, Provo, UT), during the social event. Thus, the recruited teams were asked to choose the date of the social event within a three-week data collection period which most likely would yield high player attendance.

Head coaches of recruited teams were informed about the purpose of the study, ethical considerations, and test procedures by telephone and email 1–4 weeks before the event. Additionally, a reminder was sent to the head coaches by text message or email 2–3 days before survey completion. Links and QR-codes to the online surveys and informational letters directed to players, parents, and coaches, were sent to head coaches the evening before the event. Because of the large sample size, geographical distances between football clubs and the possibility of data collection happening in two or more clubs simultaneously, the research team was not physically present at the social team events. However, a research assistant was available by phone if needed.

At the event, the head coach informed the players about the study in accordance with information sent from the research group. Thus, players were informed about the purpose of the study, the procedures, and that the survey was anonymous and participation voluntary. To minimize the risk of social desirability bias, players were instructed to answer honestly and to not speak with teammates while completing the survey. If the players had questions during the survey completion, they were instructed to ask their head coach for help. The link to the online survey was distributed to the players from the head coach by online social platforms or by a printed QR-code linking to the survey. Players used their mobile phones or computers when responding to the survey.

The head coach sent the written information, test procedures, ethical considerations, and survey links digitally by mail or social platforms to the players' parents the same day as players completed the survey. Both parents were encouraged to participate. Because early data revealed most of the participants were mothers, coaches were asked to send a reminder to parents after 1–3 days, especially encouraging fathers to participate in the survey.

### 2.3 Measures

All three respondent groups (players, coaches, and parents) were asked to indicate their gender, defined here as self-identified categories (male/female/rather not say), along with their age and whether their club had quality club status. Players reported the gender of their head coach(es), the parents specified if they were answering on behalf of a son or a daughter, and coaches indicated the gender of their team, the duration of their coaching tenure, and their coaching education.

Players' motives for participation and ambitions were measured for all groups, adapting the phrasing of the questions to suit each respondent group.

#### 2.3.1 Motives for participation

Motives for participation were assessed by asking participants to rank five pre-defined motives from 1 (most important) to 5 (least important): 1) To socialize with friends, 2) Because it is fun, 3) To develop as a football player, 4) To become a professional football player, and 5) Because parents decided. The motives related to socializing and having fun were included based on previous research showing these as key motives for both males and females participating in sports (13). Motives related to “develop as a player” and to “become a professional” were included due to known gender differences in these motivations (7). The fifth motive, “parents' decision” was included based on pre-study conversations with parents and coaches, suggesting it as a potential reason to participate. Players were asked to respond in the first person, while parents and coaches were asked to rank the motives based on their perceptions of the players' motives.

In addition to current motives to participate, we asked players and their parents to recall the most important reason why the player first started participating in football. We deemed this retrospective question irrelevant to the coaches. Response options were: “Because of football interests”, “Because parents decided”, “Because friends were participating”, “Because a parent acted as coach”, “Do not know/remember”. We included this question to uncover whether parents and players construe past motives to participate differently.

#### 2.3.2 Ambitions

Athletic ambitions were assessed by measuring the prospects of becoming a professional football player. On a Likert scale from 1 (“not at all”) to 5 (“to a high degree”), players answered how strongly they dream about becoming a professional football player, and how likely they think it is that they will become a professional (from 1 = “not likely” to 5 = “very likely”). Parents answered how strongly they *think* their youth is dreaming, and they made their own assessment of the likelihood that their youth will become a professional. We asked coaches to answer “yes” or “no” to whether they think “most of your players dream about becoming a professional”, and they used the 5-point Likert scale to evaluate the likelihood that “one or more of your players will become a professional”.

To further assess ambitions, we measured w*illingness to train*. We asked all groups to indicate their own preferred number of organized football practices per week if they could choose, using a 6-point scale ranging from 1 to more than 5 practices per week. Participants were also asked to indicate the current number of organized practices offered per week using the same scale, allowing us to test for discrepancies between actual and desired practices frequencies.

The extent to which players are practicing outside the organized activities may also serve as an indication of athletic ambitions. Therefore, we included a measure of the frequency of unorganized football practices. We asked all respondents to indicate the frequency of the players' engagement in unorganized training during the past six months. Responses alternatives were “Daily”, “More than once e a week”, “Once a week”, “1–3 times per month”, “Less often than once per month” and “Never”. We also asked about the players' desire to play unorganized football more often than they currently do. Response alternatives were “Yes”, “No” and “I do not know”.

### 2.4 Data analysis

Because the dataset includes rank-ordered responses (e.g., ranking of motives), categorical responses (e.g., recall of main motive), and Likert-type outcomes (e.g., dreams and perceived probability of becoming professional), we used descriptive summaries and inferential tests appropriate to each data type.

Descriptive summaries of our findings are reported as follows. For motive ranking, we report mean ranking scores (±SD) to summarize each motive's relative priority within each group, and we additionally report the percentage (n) of respondents assigning each motive the top rank (rank = 1). The categorical variable (recall of main motive for initially starting to participate) is presented as percentages and frequencies, and the Likert-type outcomes (e.g., dreaming and probability of becoming a professional) are presented as means and standard deviations.

To analyze whether the three groups rank the five pre-defined motives for participation differently, we used a PERMANOVA (permutational multivariate analysis of variance), a non-parametric alternative to MANOVA. This approach is appropriate because the rank-outcome is an ordinal response where ranks are not interval-scaled. PERMANOVA derives inference through permutations, thereby avoiding multivariate normality and homogeneity of covariance assumptions that are difficult to justify for rank data. Analyses were conducted for male and female athletes separately to investigate whether the potential discrepancies between groups are different across gender. We report adjusted *p*-values to decrease the risk of Type 1 error in multiple pairwise tests. To test ranking differences between male and female athletes, we used a Kruskal–Wallis test, which does not assume interval scaling of the ranks and is suitable for comparing ordinal distributions.

We used Chi-square tests to assess differences between groups on categorical variables (e.g., main motive for initially starting to play football; yes/no dreaming among coaches; frequency categories of unorganized practice).

We assessed the difference between players and their parents with regards to the Likert-type variables (e.g., “dreaming of becoming professional”) by an independent-samples *t*-test, for male and female players separately. Since coaches answered this question by a yes or no answer, we tested differences by a chi-square test. For probability of becoming professional, all three groups responded on the same 5-point Likert scale, and we analyzed differences using a one-way ANOVA with Tukey *post hoc* comparisons.

Alignment of ambitions, measured by the number of organized practices that the groups would prefer if they could choose, was tested using a one-way ANOVA with Tukey *post hoc* tests, because this preference was captured on an ordered multi-category scale that can be approximated as continuous at the sample level.

Alpha level was set to 0.05, and for mean-difference comparisons, effect sizes were assessed using Cohen's d (interpreted using conventional benchmarks). To ensure anonymity of the participants, analyzing and linking individual responses was not possible.

## 3 Results

### 3.1 Motives for participation

“Because it is fun” was the most important motivation for playing football for both genders, with 40% of the female players and 37.4% of the male players ranking this as number one (for details see Table 1). The parents of the players and the coaches of male players also ranked “Because it is fun” as number one, while coaches of female players ranked “To socialize with friends” as number one. Both genders ranked “To develop as a football player” as number two, while “Parents decision” was ranked last for both genders. The parents of the players and the coaches of male players ranked “To socialize with friends” as number two, and the coaches of female players ranked “Because it is fun” as number two.

**Table 1:** Ranking of motivations to play football across groups (1 = most important, 5 = least important). Data are presented as mean ranking score and standard deviation (SD), and as percentages (%) and number (*n*) of top ranking.

Respondent group	Fun	Socializing	Development	Professional	Parents' decision
Female players (*n* = 265)	**2.02** (1.07)	2.57 (1.21)	2.30 (0.98)	3.60 (1.18)	4.52 (0.87)
	**40.0%**	25.7%	25.3%	7.2%	1.9%
	(*n* = 106)	(*n* = 68)	(*n* = 67)	(*n* = 19)	(*n* = 5)
Male Players (*n* = 321)	**2.13** (1.08)	2.68 (1.23)	2.37 (0.98)	3.27 (1.327)	4.55 (0.95)
	**37.4%**	23.7%	21.2%	14.6%	3.1%
	(*n* = 120)	(*n* = 76)	(*n* = 68)	(*n* = 47)	(*n* = 10)
Coaches for females (*n* = 46)	1.91 (0.96)	**1.87** (1.09)	2.70 (0.87)	4.22 (0.87)	4.30 (0.87)
	39.1%	**47.8%**	8.7%	2.2%	2.2%
	(*n* = 18)	(*n* = 22)	(*n* = 4)	(*n* = 1)	(*n* = 1)
Coaches for males (*n* = 47)	**1.60** (0.80)	2.00 (0.86)	2.66 (0.84)	4.13 (0.85)	4.62 (0.53)
	**57.4%**	34.0%	6.4%	2.1%	0.0%
	(*n* = 27)	(*n* = 16)	(*n* = 3)	(*n* = 1)	(*n* = 0)
Parents to females (*n* = 197)	**1.80** (0.88)	2.04 (1.10)	2.65 (0.85)	4.16 (0.91)	4.35 (0.91)
	**43.7%**	41.6%	11.2%	1.5%	2.0%
	(*n* = 86)	(*n* = 82)	(*n* = 22)	(*n* = 3)	(*n* = 4)
Parents to males (*n* = 305)	**1.91** (0.89)	2.14 (0.89)	2.53 (0.95)	3.88 (1.09)	4.54 (0.83)
	**38.7%**	35.4%	18.4%	6.2%	1.3%
	(*n* = 118)	(*n* = 108)	(*n* = 56)	(*n* = 19)	(*n* = 4)

A lower mean ranking score indicates higher importances. Percentages indicate frequency of top-ranking (1). Highest mean score/frequency of top rating for each group in bold.

There were significant differences in motives for participation in football between players, coaches, and parents, for both male (*F* = 13.54, *p* < .01) and female players (*F* = 10.76, *p* < .01). These findings indicate that the way motives for participation were ranked varied significantly depending on group membership. In the following, we report *post hoc* analyses for differences between players and their coaches and parents. To facilitate interpretation of these results, we provide mean ranking scores across all groups, along with the percentage of individuals in each group who rated each motive as the most important (see Table 1).

#### 3.1.1 Social motive

For male players, coaches (*F* = 13.31, *p* < .01) and parents (*F* = 33.85, *p* < .01) ranked social motives significantly higher than the male players themselves. Similar results were reported for female players, where both coaches (F = 13.47, *p* < .01) and their parents (F = 23.12, *p* < .01) ranked social motives higher than the players. Whereas only 25.7% of the female players rated social motives as the most important reason for playing football, 47.8% of their coaches and 41.6% of their parents considered this the top motive for the players. In comparison, 23.7% of male players ranked social motives as their top motive, compared to 34% of their coaches and 35.4% of their parents. There were no significant differences between male and female players on their own rankings of social motives (H(1) = 1.311, *p* = .252).

#### 3.1.2 “Having fun” motive

For male players, coaches (*F* = 18.31, *p* < .01) and parents (*F* = 18.31, *p* < .05) ranked the motive “Because it is fun” significantly higher than the players themselves. For female players there were no significant differences between the female players and their coaches, and only a marginally differences between the female players and their parents (*F* = 5.21, *p* = .06). Examining the mean ranking scores and percentages of top rating (see Table 1), there was an indication that parents reported “Because it is fun” slightly higher than the players themselves. There was no significant difference between male and female players on their rankings of “having fun” as a motive to play football (H(1) = 2.139, *p* = .144).

#### 3.1.3 Development motive

For male players, there were no significant differences between the players and their coaches and parents on the ranking of “To develop as a football player” as the motive to play football. However, female players ranked “to develop as a football player” significantly higher than both their coaches (*F* = 6.62, *p* < .05) and their parents (*F* = 16.14, *p* < .01). There was no significant difference between male and female players on their rankings of the development motive (H(1) = .666, *p* = .414).

#### 3.1.4 Professional motive

The male players ranked “To become a professional football player” significantly higher than both their coaches (*F* = 18.31, *p* < .01), and their parents (*F* = 38.22, *p* < .01). Similar results were found for female players where “To become a professional football player” was ranked significantly higher by the female players compared to their coaches (*F* = 11.69, *p* < .01), and their parents (*F* = 31.53, *p* < .01). “To become a professional football player” was the only motive for which there was a significant difference between male and female players (H(1) = 8.168, *p* < .01), and the male players ranked this motive higher than the female players (see Table 1).

#### 3.1.5 Parents' decision motive

There were no significant differences between any of the groups regarding the rating of the “parents decided” motive. All groups ranked this motive as the least important for playing football. There was no significant gender difference between players on this motive (H(1) = 1.407, *p* = .236).

### 3.2 Recall of motive for initially starting to play

Both male (62.9%) and female (47.9%) players recalled “football interests” as the most important motive to initially start playing football, followed by “friendships” (i.e., social motive) (see Table 2). Parents to the male players also recalled “football interests” (59%), as the most important motive to initially start playing football, while parents to female players recalled friendship (49.2%) as the most important motive why they initially started playing football.

**Table 2:** Recall of main motives for starting to participate in organized football. Data are presented as percentage (%) and number of participants (*n*).

Respondent group	Interest	Friendship	Parents' decision	Parents were coaches	Don't know
Female players (*n* = 265)	**47.9%** **(127)**	36.6% (97)	5.7% (15)	4.2% (11)	5.7% (15)
Male players (*n* = 318)	**62.9%** **(200)**	25.5% (81)	6.3% (20)	1.9% (6)	3.5% (11)
Parents to females (*n* = 214)	31.5% (62)	**49.2%** **(97)**	10.7% (21)	5.1% (10)	3.6% (7)
Parents to males *n* = 305)	**59.0%** **(180)**	26.6% (81)	8.2% (25)	4.6% (14)	1.6% (5)

Main motive most frequently selected for each group in bold.

Although the order of recalled motives was consistent across genders, there were significant differences in the relative frequencies (*χ*^2^(4) = 15.848, *p* < .01). A significantly higher proportion of females (36.6%) compared to males (25.5%) recalled starting to play football due to social motives, while significantly more males (62.9%) than females (47.9%) recalled starting due to a genuine interest in football.

There were no significant differences between the parents of male players and the male players themselves in the recall of the players primary motive for starting to play football (*χ*^2^(4) = 6.79, *p* = .147). In contrast, parents of female players differed significantly from the female players (*χ*^2^(4) = 16.664, *p* = .002). The parents of the female players reported the social motive most frequently as the primary motive (49.2%), whereas only 34.5% recalled “interest in football” as the female players' initial motive. These findings suggest that female players were more likely to recall interest in football as their primary motive for starting to play than their parents remembered.

### 3.3 Athletic ambitions

Male players (M = 3.59, SD = 1.27) reported *dreaming* about becoming a professional player significantly more compared to their parents' beliefs (M = 2.67, SD = 1.67, *p* < 0.001, *d* = 0.625). Similarly, female players (M = 3.20, SD = 1.27) reported higher levels of dreaming about becoming a professional player compared to their parents (M = 2.02, SD = 1.42, *p* < 0.001, *d* = 0.882). The underestimation of dreaming was more pronounced for females, as indicated by the larger effect size. Male football players scored significantly higher than the female football players on *dreaming* about becoming a professional player (*p* < .001, *d* = 0.310).

Coaches for males (37.4%) were significantly more likely to believe that most of their players dreamed about becoming professional players compared to coaches for females (9.8%, *χ*^2^(1) = 10.64, *p* < 0.01).

When evaluating beliefs about the *likelihood* of becoming a professional player, mean scores differed significantly across all six groups (*F*(5, 1,214) = 6.89, *p* < 0.001). Male players (M = 2.42, SD = 1.13) believed their chances were significantly higher than their parents' beliefs (M = 2.08, SD = 0.98, *p* < 0.001). There were no significant differences between the male players and the coaches (M = 2.42, SD = 1.15, *p* = 0.98). Female players (M = 2.18, SD = 1.00) also perceived their chances as higher than their parents did (M = 1.97, SD = 0.95, *p* < 0.05). However, their beliefs were slightly, but not significantly lower than those of their coaches (M = 2.45, SD = 1.03, *p* = 0.08). There were no significant gender differences in players' beliefs of becoming professional players (*p* = .055).

Alignment of ambitions, measured by the number of organized practices preferred by the groups, differed significantly across group membership (*F*(5, 1,213) = 19.578, *p* < .001) (Table 3). Players reported preferring a higher number of practices than their parents did, with this misalignment evident for both males (MD = 0.43, *p* < .001) and females (MD = 0.70, *p* < .001). Parents preferred fewer practices for their daughters compared to their sons (MD = 0.27, *p* = .023), highlighting a gender-related difference in parents' preferences.

**Table 3:** Number of organized and preferred organized football training sessions pr week, reported as mean score on a 6-point scale and standard deviations (SD).

Respondent group	Organized football training sessions	Preferred organized football training sessions
	M (SD)	M (SD)
Female players (*n* = 268)	2.85 (0.92)	3.57 (1.11)
Male players (*n* = 325)	3.05 (0.94)	3.54 (1.12)
Coaches for females (*n* = 46)	2.59 (0.75)	3.12 (0.65)
Coaches for males (*n* = 47)	2.87 (0.73)	3.35 (0.84)
Parents to females (*n* = 197)	2.80 (0.68)	2.85 (0.73)
Parents to males (*n* = 305)	3.06 (0.93)	3.12 (0.93)

While males' preferences were in alignment with their coaches, females preferred a higher number of practices than their coaches recommended (MD = 0.45, *p* < .05). However, there was no significant gender difference among players themselves, as males and females reported preferring the same number of organized practices. However, when examining the actual number of practices reported by the players, female teams had fewer practices than male teams, although this difference was only marginally significant (MD = 0.19, *p* = 0.077).

Finally, ambitions were also assessed by the frequency of unorganized practices, as reported by players and their parents. Male players reported engaging in unorganized training more frequently than female players, with 60.7% of males stating they train every day or multiple times per week compared to 33.2% of females (*χ*^2^(5) = 63.798, *p* < 0.001). This pattern aligns with the perceptions of their parents.

When asked whether they would like to engage more frequently in unorganized training, 67.4% of males and 71.6% of females indicated they would, suggesting that males and females are equally ambitious in their desire to train more often outside of organized practices. However, more parents believed their sons (58.9%) have a desire to engage more in unorganized practices compared to their daughters (47.2%, *χ*^2^(2) = 7.731, *p* < .05).

## 4 Discussion

While prior research has examined the influence of parents and coaches on athletes' motivation (29, 30), the present study extends this literature by examining and identifying misalignment between players and significant socializers (i.e., coaches and parents), as well as the gendered biases associated with these misalignments.

In accordance with our first hypothesis, the results revealed significant misalignments between the beliefs held by parents and coaches and the actual ambitions and motives of youth football players. Specifically, parents and coaches tend to overestimate the importance of social interaction and enjoyment as motives for participation, while underestimating players' development motives and dreams of becoming professional athletes.

One possible explanation for this misalignment lies in dominant cultural frameworks within Norwegian sport. The vision of “Joy of sports for all” in Norwegian sport emphasises participation, enjoyment, and inclusivity over competition and elite performance (31). Among adults, adherence to this norm is widely expected, and strong expressions of ambition or elite orientation may be perceived as socially inappropriate. This tendency may be further reinforced by broader cultural norms associated with the “Law of Jante’, which discourage overt self-promotion and the articulation of exceptional aspirations (32). Together, these norms may shape how adults interpret and evaluate young athletes' ambitions, leading them to frame sport primarily as a recreational and developmental arena rather than a pathway toward professional performance. Youth, by contrast, may not yet have fully internalised these cultural expectations, potentially contributing to divergent perceptions of what sport participation can or should entail.

Importantly, the assumption that a strong performance orientation necessarily undermines enjoyment is not consistently supported by empirical evidence. For example, a study examining sources of enjoyment in sport among Brazilian female volleyball and basketball players found no significant association between a high aggregated amount of deliberate practice and reduced enjoyment, despite “having fun” being reported as the primary motive for participation (33). Moreover, substantial individual differences were observed in how players defined and experienced enjoyment in sport. This suggests that “having fun” may be interpreted differently by players and adults: whereas adults may associate enjoyment primarily with social interaction, some young athletes may experience enjoyment through performance-oriented training and skill development. Nevertheless, the present study does not directly examine how “fun” is defined by players or adults, and future research should therefore explore variations in perceptions of enjoyment in sport.

The misalignments between players and significant socialising agents documented in our research are important for both coaches and parents to recognise. Misconceptions regarding players' motives for participation may lead adults to unintentionally encourage or constrain sport participation in ways that are misaligned with athletes' own goals, thereby increasing the risk of dropout. If mismatches in motivational alignment do, in fact, reduce enjoyment and contribute to sport attrition, addressing such discrepancies becomes relevant not only from a sporting perspective but also from a public health standpoint. Given that positive sport experiences during youth are associated with a higher likelihood of sustained physical activity and better health in adulthood (34), reducing motivational misalignment, and consequently dropout, may represent a meaningful strategy for counteracting the growing prevalence of sedentary behaviour and its associated health consequences.

Our second hypothesis predicted that the misalignment would be more pronounced in female players. While our data shows that parents and coaches share similar misconceptions about female and male players' motives to participate, some of our findings support the prediction that females' ambitions are particularly underestimated by parents and coaches. One notable gendered misalignment is the coaches' overestimation of girls' social motivations, and their underestimation of development-motives. Although socializing with friends is indeed an important motivation for female youth athletes, there is also a prominent desire for competitiveness and skill development, which tends to be neglected by coaches (12). Such biased gender beliefs may explain why coaches often adopt different coaching styles depending on whether they coach males or females (35). Derived from a cultural understanding of gender differences, it is generally more acceptable to use an aggressive, performance-oriented coaching style for boys than for girls (1).

Following the same pattern, parents underestimated their daughters' motivations to develop skills, to engage in both organized and unorganized training, and to dream of becoming professional, a misalignment that was not as pronounced for male players. Also, parents underestimated the interest in football as the reason why their daughters started to play football, while the same misalignment was not seen toward their sons. This pattern where adults overemphasize the social aspects of sports participation may downplay the developmental and performance aspects for girls. This may lead to a scenario where female players receive fewer training sessions and lower quality training that prioritizes social aspects over skill development. We do not assert that adults' preference for less frequent training for girls is a conscious intent to provide them with inferior opportunities, but rather a form of “discrimination of low expectations” that may be unconscious. The potential consequences of gender biased beliefs among parents and coaches are significant, as females may be provided with less rigorous training programs and less access to high-quality coaching, and may drop out from football.

According to Eccles' Expectancy-Value Theory (14), parents' and coaches' beliefs and values may shape young athletes' self-concept, and over time, shaping their ambitions and behaviors for becoming a professional athlete. This theoretical framework may also help explain why both the present study and previous research (1, 2) find that males report higher ambitions than females of becoming professional football players. If significant socializers hold beliefs, values, and behaviors that implicitly favor males as professional athletes, these socialization processes may shape male and female players' self-concept differently. This may explain why the girls in our study were less likely to envision themselves as future professional football players and potentially reinforcing stereotypes that female athletes are less ambitious. As a consequence, female athletes may adjust their behavior, for example by dreaming less, taking training less seriously, or drop out from sport.

Coaches and parents play a critical role in creating motivational climate (14, 36), and in this endeavor, it is crucial that they are aware of their own potential behavioral biases influenced by gender-stereotypes. If parents and coaches underestimate their child's ambitions, or misinterpret their main motivations, they may unconsciously project traditional gender roles onto their child, reinforcing the gender differences in sport. In the long run, the reinforcement of stereotypical gender roles may limit female opportunities not only to become professional athletes, but also to pursue traditionally male-dominant roles in sport, such as coaching or leadership positions.

Unconscious gender bias in the misalignment of parents, coaches and players motives may also reinforce the disparities in resource allocation between female and male youth football players, as experienced by Norwegian female youth players (1). Identifying gender bias in perceived motives, and increasing awareness of such bias among significant socializers, may therefore be an important step toward gender equity, which in turn may support sustained participation in football and sports.

Addressing gendered misalignments requires increasing awareness among parents and coaches of the potential impact of their gendered expectations. Providing accurate and supportive feedback to both male and female players is essential to fostering motivation and equal opportunities in sport. This could be achieved through mandatory coaching courses, systematic focus within football clubs, and guidance for parents. In addition, clarifying expectations between coaches and players, between parents and players, and between parents and coaches may be important to align training and everyday routines with the ambitions of each player.

Some limitations of the current study should be acknowledged. Although players and coaches completed the survey in a controlled setting, were assured of anonymity, and were encouraged to respond honestly and independently, we cannot rule out that some responses reflected perceived expectations rather than participants' true views (i.e., social desirability bias). Similar concerns apply to parents, who completed the questionnaire in an uncontrolled setting and may have consulted others while responding. Nonetheless, the large sample size strengthens the robustness and generalizability of our findings. Future research could complement questionnaire-based design with alternative approaches, such as in-depth qualitative interviews, to mitigate these limitations and provide a richer, more nuanced understanding of how gender bias is experienced.

Finally, although misalignment may be an important factor in understanding sport participation and gendered expectations, we acknowledge that these issues emerge through complex and multifaceted processes (33). Misalignment should therefore be considered alongside other relevant individual, social, and structural factors when designing and implementing interventions to promote sustained and equitable sport participation.

## Data Availability

The raw data supporting the conclusions of this article will be made available by the authors, without undue reservation.
